# Temporal effects of disturbance on community composition in simulated stage‐structured plant communities

**DOI:** 10.1002/ece3.3660

**Published:** 2017-11-23

**Authors:** Youshi Wang, Shujun Wen, M. D. Farnon Ellwood, Adam D. Miller, Chengjin Chu

**Affiliations:** ^1^ SYSU‐Alberta Joint Lab for Biodiversity Conservation Department of Ecology State Key Laboratory of Biocontrol and School of Life Sciences Sun Yat‐sen University Guangzhou China; ^2^ Guangxi Key Laboratory of Plant Conservation and Restoration Ecology in Karst Terrain Guangxi Institute of Botany Guangxi Zhuang Autonomous Region and Chinese Academy of Sciences Guilin China; ^3^ Centre for Research in Biosciences University of the West of England Bristol UK; ^4^ Conservation Ecology Center Smithsonian Conservation Biology Institute National Zoological Park Front Royal VA USA; ^5^ Institute for Sustainability, Energy, and Environment University of Illinois at Urbana‐Champaign Urbana IL USA

**Keywords:** compositional change, grid‐based model, individual‐based model, neutral model, stage structure

## Abstract

In an era of global environmental change, understanding how disturbance affects the dynamics of ecological communities is crucial. However, few studies have theoretically explored the potential influence of disturbance including both intensity and frequency on compositional change over time in communities with stage structure. A spatially explicit, individual‐based model was constructed incorporating the various demographic responses to disturbance of plants at two different growth stages: seedlings and adults. In the model, we assumed that individuals within each stage were demographically equivalent (neutral) but differed between stages. We simulated a common phenomenon that seedlings suffered more from disturbance such as grazing and fire than adults. We showed how stage‐structured communities of seedlings and adults responded to disturbance with various levels of disturbance frequency and intensity. In “undisturbed” simulations, the relationship between average species abundance (defined here as the total number of individuals divided by species richness) and community composition turnover (measured by the Bray–Curtis similarity index) was asymptotic. However, in strongly “disturbed” simulations with the between‐disturbance intervals greater than one, this relationship became unimodal. Stage‐dependent response to disturbance underlay the above discrepancy between undisturbed and disturbed communities.

## 1 INTRODUCTION

Understanding the increasingly severe impacts of natural and anthropogenic disturbance on the structure of communities is critical to conserve and manage what is left of the earth's natural biodiversity (Dornelas, 2010; Magurran, 2007). The precise mechanisms governing the relationship between disturbance and species richness, that is, the intermediate hypothesis remain controversial (Fox, 2013; Sheil & Burslem, 2013), meanwhile the undeniable impacts of high levels of disturbance on species richness are becoming increasingly well documented (Bazzaz, 1996; Bunn, Jenkins, Brown, & Sanders, 2010; Connell, 1978; Hughes, Byrnes, Kimbro, & Stachowicz, 2007; Kadmon & Benjamini, 2006; Zhang, Mayor, & He, 2014).

The word *disturbance* is associated with ecological succession and often used generally, relating to any one of a range of specific phenomena such as the effects of fire, storms, or animal grazing on plants. From a theoretical perspective, all these sources can be unified under the concept of disturbance, because they eventually lead to modification of the fundamental demographic processes of individuals such as birth, death, and immigration (Dornelas, 2010; Kadmon & Benjamini, 2006). These fundamental processes do more than govern the number of individuals per species (population growth); local extinctions and immigration govern the number of species per community (species richness), and the proportional abundance of each of those species. Besides species richness, we believe that evaluating temporal variations of community composition is fundamental to understand the essential biological mechanisms underlying population and community dynamics, which is another crucial dimension of biodiversity (Debussche, Escarré, Lepart, Houssard, & Lavorel, 1996; Pickett, Collins, & Armesto, 1987).

In undisturbed plant communities, decreasing average species abundance (ASA), defined as the total number of individuals divided by species richness, increases the average risk of local extinction through demographic stochasticity (Kadmon & Benjamini, 2006; McGlynn, Weiser, & Dunn, 2010; Srivastava & Lawton, 1998). When ASA is small, communities experience a rapid turnover of species whether the community is saturated by individuals or not. It follows that when ASA is large, compositional turnover will slow down as communities reach saturation in terms of individuals. In this case, immigration from the regional species pool would contribute little to the composition of communities. For instance, our previous work revealed an asymptotic relationship between ASA and the rate of compositional turnover (Wang et al., 2013). This implies that ASA could serve as a tractable and effective way of quantifying temporal effects of disturbance on community composition. Identifying a simple, robust parameter that can quantify the compositional turnover of communities will inform protocols for the conservation of biodiversity and land management.

In disturbed plant communities, increased mortality caused by disturbance reduces the total individuals of communities (Dornelas, 2010; Sousa, 1984). Under these conditions, demographic stochasticity leads to increased species turnover through time. It therefore makes intuitive sense that strongly disturbed communities should experience faster rates of species turnover than less disturbed ones. But disturbance also prevents communities from reaching saturation in terms of individuals, releasing available resources to locally produced offspring and immigrants from the regional species pool, which will slow temporal turnover of communities (Dornelas, 2010; Kadmon & Benjamini, 2006). So far, how these two opposite processes jointly determine the community temporal turnover and whether ASA could indicate temporal turnover remain unclear in disturbed communities.

To theoretically explore how disturbance influences community temporal dynamics, we constructed a stage‐structured, spatially explicit individual‐based model (IBM), in which every individual was labeled and the fundamental demographic processes determined the behavior of the communities. We modeled disturbance via manipulation of the mortality rates of seedlings rather than adults, thus incorporating the responses of different growth stages into the model (Decocq, Beina, Jamoneau, Gourlet‐Fleury, & Closset‐Kopp, 2014; Suresh, Dattaraja, & Sukumar, 2010). In the model, individuals within each stage were demographically equivalent (neutral) but differed between stages. We focused on how communities responded to a single disturbance event, as other studies did (Dornelas, 2010). We used simulation experiments to answer the following two critical questions: (1) How disturbance intensity and frequency jointly influence community compositional change over time, and (2) Whether ASA could reflect the temporal turnover of composition in disturbed communities. Unless stated otherwise, we limited the application of ASA to indicate compositional change for communities with shared regional species pool.

## 2 MATERIALS AND METHODS

The model description follows the ODD (overview, design concepts, details) protocol for describing IBMs (Grimm et al., 2006, 2010). Simulation experiments were implemented using NetLogo software (v5.0.4) (Wilensky, 1999).

### 2.1 Purpose

A stage‐structured, spatially explicit IBM was developed to explore the effect of disturbance (frequency and intensity) on the compositional change in stage‐structured communities.

### 2.2 Entities, state variables, and scales

#### 2.2.1 Individuals

An individual in the model is characterized by the following state variables: species identity, spatial coordinates, birth rate (*b*), death rate (*m*), and age. Whether an individual gave rise to offspring depends on its age comparing to the threshold of maturity. For the sake of computation time, here we set reproductive maturity to 3 years (The time of reproductive maturity did not qualitatively influence the simulation behavior).

#### 2.2.2 Temporal resolution

One time step in simulation experiments represents 1 year, with the 1‐year increase of age for survived individuals.

### 2.3 Process overview and scheduling

Our model explicitly incorporates fundamental demographic processes, executed in order of *Birth and Dispersal* of offspring, *Immigration* (IM) from regional species pool (*S*), and *Death*. These processes were described in detail in the section 2.7 for submodels.

### 2.4 Design concepts

#### 2.4.1 Basic principles

Neutral models of biodiversity have been extensively used in previous studies to investigate local community dynamics (Bell, 2001; Hubbell, 2001) and the effect of disturbance on species richness (Kadmon & Benjamini, 2006). The model developed here is an extension of these models by incorporating stage‐dependent demographic responses to disturbance.

#### 2.4.2 Interaction

Indirect competition for empty space exists among individuals; that is, offspring and immigrants could only occupy the empty cells.

#### 2.4.3 Stochasticity

There are two stochastic components in the model: (1) the random spatial distribution of the individuals at model initialization and (2) the random spatial location of empty cells recruited by the immigrants from regional species pool.

#### 2.4.4 Observation

The species composition of communities after a single disturbance event until the next disturbance was recorded.

### 2.5 Initialization

We set up a landscape where the local community consists of *A* cells. The initial landscape was saturated, being occupied by all species present in the regional species pool, and with the number of individuals for each species following a log‐series distribution (Keddy, 2005; Wang et al., 2013). To avoid edge effects, a “wraparound” approach (i.e., periodic or toroidal boundary conditions) was used (Chave, Muller‐Landau, & Levin, 2002).

### 2.6 Input data

The model does not use input data to represent time‐varying processes.

### 2.7 Submodels

#### 2.7.1 Birth and Dispersal

Every adult produces propagules at the rate *b*, which are classified as seedlings. Seedlings cannot produce offspring. We incorporated an intermediate type of dispersal of offspring into the simulations, this being a compromise between the extreme dispersal modes of local and global dispersal. Under this scenario, we assume that newborn offspring disperse according to a dispersal kernel taking the following form (Clark, Silman, Kern, Macklin, & HilleRisLambers, 1999):

$$K_{2\mathrm{Dt}}\left(r\right)=\frac{2\;pr}{u{\left[1+\left(r^{2}/u\right)\right]}^{p+1}},p>0$$

where *u* and *p* are parameters determining the shape of the function. This kernel combines Gaussian dispersal at short distances with a power‐law tail of long‐distance dispersal (Chave et al., 2002; Clark et al., 1999).

#### 2.7.2 Immigration

To ensure that our model was biologically realistic, we accounted for the immigration of species from a regional species pool. The species abundance distribution for species pool was set to follow a log‐series distribution (Gravel, Canham, Beaudet, & Messier, 2006). At each time step, a fixed number *IM* (immigration rate) of seedlings (equal to or less than *IM* species) were randomly drawn with replacement from the species pool, and globally dispersed at random into the local community. Both dispersing offspring and immigrants could only colonize vacant sites.

#### 2.7.3 Death

Having set *m*_young_ and *m*_old_ as the intrinsic mortality rates for seedlings and adults, we set *m*_young_ at a higher level than *m*_old_, to account for the fact that seedling mortality usually exceeds that of adults (Suresh et al., 2010). In our simulations, we set *m*_young_ at 1.2 times greater than *m*_old_.

Although different definitions of disturbance have been proposed, one fundamental outcome of disturbance is the increased mortality of individuals (Dornelas, 2010; Sousa, 1984). For instance, some empirical studies have demonstrated that seedlings are more sensitive to environmental change/disturbance than adults (Decocq et al., 2014; Sukumar, Suresh, Dattaraja, Srinidhi, & Nath, 2005; Suresh et al., 2010). Such differential responses of different demographic stages to disturbance are ubiquitous in nature (Decocq et al., 2014; Green, Harms, & Connell, 2014; Suresh et al., 2010). To incorporate this feature into the model, we assume that disturbance increases the mortality rate of seedlings but has ignorable effect on adults. This assumption mimics well various types of disturbance events in natural communities, including the grazing and fire in forests (Edwards & Krochenberger, 2006; Sukumar et al., 2005). Thus, the seedling mortality rate under disturbance is *D *× *m*_young_, where *D* represents disturbance intensity. *D *=* *1 means that no disturbance occurs. Seedlings in the simulated community have identical mortality rates (*D *× *m*_young_), and adults have identical birth rates (*b*) and mortality rates (*m*_old_).

Each individual experienced a corresponding risk of mortality, depending on the stage the individual belongs to. By comparing the realized mortality rate of seedlings (*D *× *m*_young_) or *m*_old_ to a random number from a [0, 1] uniform distribution, we determined the fate of the focal individual in terms of its survival; when effective mortality is greater than the random sample, then the individual is killed.

### 2.8 Simulation experiments and data analysis

We conducted a set of factorial simulations within the following parameter space: birth rate *b* for all species ranges from 0.1 to 2.7 with the interval 0.2; death rate of adults *m*_old_ = 0.05, 0.1, and 0.15; immigration rate *IM* = 20 and 60. We simulated three levels of disturbance intensity for each parameter setting: *D *=* *1.0, 3.0, and 5.0. Model parameters, their meanings, and values taken are summarized in Table 1. All simulations for undisturbed communities were run for 10,000 time steps in order to allow communities to reach a dynamical equilibrium state in terms of species richness. We set identical simulation time for communities suffering from disturbance.

**Table 1:** Model parameters, their meanings, and values taken

Parameter	Meaning	Values
*A*	Landscape size	100 × 100 cells
*S*	Regional species pool	200 species
*IM*	Immigration rate	20 and 60 individuals
*b*	Birth rate	0.1~ 2.7, with the interval of 0.2
*m*_old_	Intrinsic death rate for adults	0.05, 0.1, and 0.15
*m*_young_	Intrinsic death rate for seedlings	1.2 × *m*_old_
*D*	Disturbance intensity	1.0, 3.0, and 5.0
*F*	Disturbance frequency	1.0, 3.0, and 5.0

**Figure 1:**
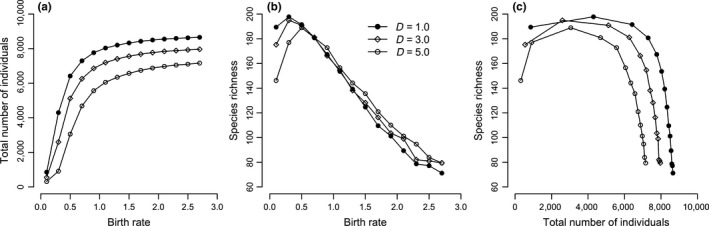
Influence of birth rate on community metrics. (a) Simulated relationships between birth rate and the total number of individuals, (b) Simulated relationships between birth rate and species richness, and (c) Simulated relationships between the total number of individuals and species richness. Three levels of disturbance intensity were explored: *D *=* *1.0, 3.0, and 5.0. *D *=* *1.0 means no disturbance. The case with the disturbance frequency equal to 2 years was presented as an example here. Immigration rate is equal to 20. Each data point represents the mean of ten replicates for each parameter combination

**Figure 2:**
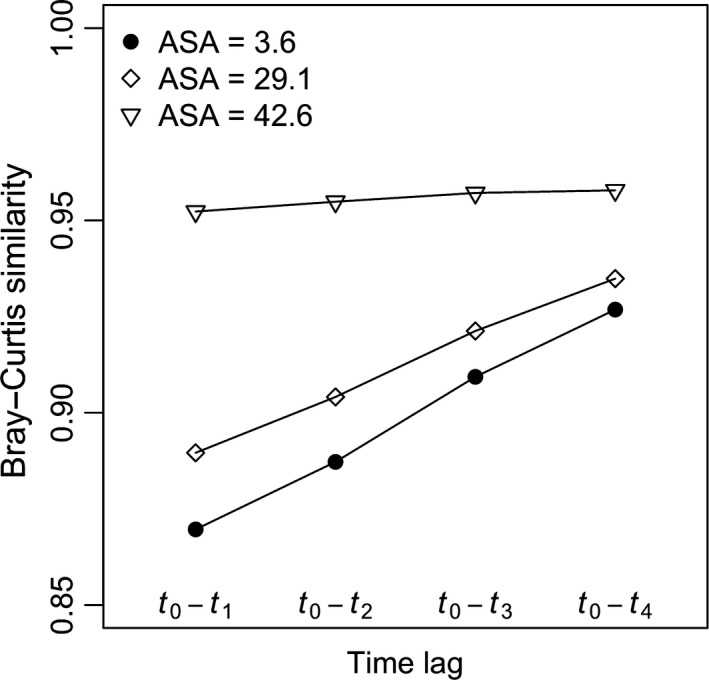
Compositional change of simulated communities over time. The case with the disturbance frequency (*F*) equal to 5 years and disturbance intensity (*D*) equal to three was presented as an example here. ASA obtained at *t*_0_ represents average species abundance determined by demographic processes as a result of disturbance. The labels of *t*_0_–*t*_1_, *t*_0_–*t*_2_, *t*_0_‐*t*_3_, and *t*_0_–*t*_4_ represent the comparisons between the disturbed community (*t*_0_) and the communities 1, 2, 3, and 4 years after a disturbance event. Immigration rate is equal to 20. Each data point represents the mean of ten replicates for each parameter combination

**Figure 3:**
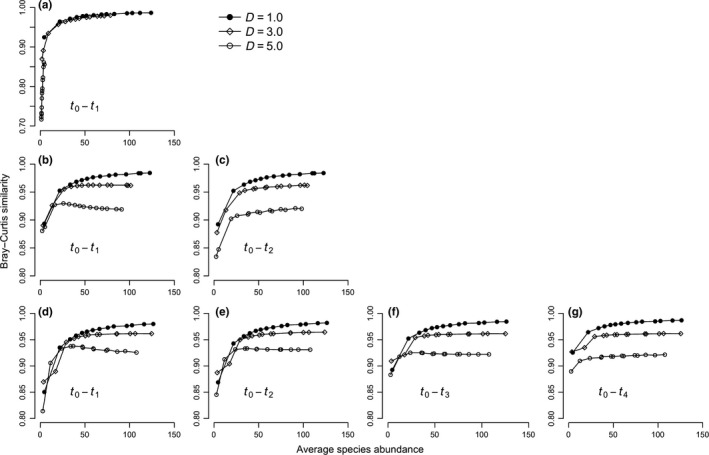
Simulated patterns between average species abundance and temporal compositional change measured by Bray–Curtis similarity (a, the disturbance frequency equal to 1 year; b–c, the disturbance frequency equal to 3 years; d–g, the disturbance frequency equal to 5 years). Three levels of disturbance intensity were explored: *D *=* *1.0, 3.0, and 5.0. The maturity time for seedlings was equal to 3 years. The labels of *t*_0_–*t*_1_, *t*_0_–*t*_2_, *t*_0_–*t*_3_, and *t*_0_–*t*_4_ represent the comparisons between the disturbed community (*t*_0_) and the communities 1, 2, 3, and 4 years after a disturbance event. Immigration rate is equal to 20. Each data point represents the mean of ten replicates for each parameter combination

We conducted a total of 7,560 simulations: 10 replicates × 3 levels of disturbance frequency (*F *=* *1.0, 3.0, and 5.0) × 3 levels of disturbance intensity (*D *=* *1.0, 3.0, and 5.0) × 2 levels of immigration (*IM* = 20 and 60) × 14 levels of birth rate (*b* is from 0.1 to 2.7 with the interval of 0.2) × 3 levels of death rate (*m*_old* *_= 0.05, 0.1, and 0.15). The results presented (Figures 1, 2, 3) are based on the setting with mortality rate 0.1, regional species pool 200, and immigration rate 60 (see Figures S2–S4 for results with the immigration rate equal to 20).

To explore the responses of communities to disturbance, we recorded the species composition of communities after a single disturbance event until the next disturbance. In addition, previous studies have demonstrated that disturbance frequency and intensity might interact to influence community structure (Miller et al. 2011; Hall et al. 2012). To this end, we conducted additional simulations with different frequencies of disturbance. In addition, due to the potential interactions between disturbance frequencies and reproductive maturity of seedlings (3 years), we set up three scenarios with the disturbance frequencies (*F*) smaller (1 year) and larger than (5 years), and equal to (3 years) the maturity, respectively. We used the Bray–Curtis similarity index (Bray and Curtis 1957) calculated in the package *fossil* (Vavrek, 2011) on the R platform (R Development Core Team 2014) to quantify compositional changes of communities across time, as this index accounts for both the incidence and the abundance of each species. The larger the Bray–Curtis similarity value, the more similar the community composition. We used the ASA, that is, the total number of individuals divided by species richness, and coefficients of variation (CV) of species abundance for the disturbed community to indicate the compositional change of this community across time until the next disturbance event. Take the scenario with the frequency with 3 years as an example. After 10,000 startup steps, we compared the compositional change between the disturbed community at the step of 10,002 (*t*_0_) and the communities followed without disturbance at the steps of 10,003 (*t*_1_) and 10,004 (*t*_2_), respectively. This is for one disturbance event. Similarly, we compared the compositional change between the disturbed community at the step of 10,005 (*t*_0_) and the communities followed without disturbance at the steps of 10,006 (*t*_1_) and 10,007 (*t*_2_). We repeated this processes until 50 disturbance events recorded, and Bray–Curtis similarity values, ASA, and CVs of species abundance were obtained by averaging individual values across 50 disturbance events and across ten replicates.

## 3 RESULTS

Simulations of undisturbed communities (the disturbance intensity *D *=* *1.0) show that increasing birth rates increase the number of individuals in the community (Figure 1a). Species richness increased slightly and then decreased sharply (Figure 1b), resulting in a hump‐shaped curve between community size (total number of individuals) and species richness (Figure 1c). Simulations of disturbed communities generated similar unimodal patterns.

The compositional change over time is presented in Figure 2 for the scenario with the disturbance frequency (*F*) equal to 5 years and the disturbance intensity (*D*) equal to three. Bray–Curtis similarity values increased with the time after disturbance, especially when ASA was low. Under no disturbance (*D *=* *1.0), asymptotic curves emerged between ASA and Bray–Curtis similarity in all three scenarios of disturbance frequency (Figure 3). When the disturbance occurred every year, the relationships between ASA and similarity displayed asymptotic patterns as well but with faster compositional change (Figure 3a). However, when the disturbance frequencies were larger than one (3 and 5 years), especially for communities suffering from strong disturbance (*D *=* *5.0), the Bray–Curtis similarity values firstly increased then decreased (Figure 3b,d). Such unimodal patterns gradually disappeared over time until the next disturbance event (Figure 3b,c for the disturbance frequency of 3 years, and Figure 3d–g for the disturbance frequency of 5 years). Relationships between CV of species abundance and Bray–Curtis similarity displayed similar patterns described above (see Figure S1).

## 4 DISCUSSIONS

As we expected, the simulated relationship between ASA and species turnover was asymptotic for undisturbed communities (Figure 3). In unsaturated communities, in line with the more individuals hypothesis, larger ASA on average reduced the likelihood of stochastic extinctions (Kadmon & Benjamini, 2006; McGlynn et al., 2010; Srivastava & Lawton, 1998). Under these conditions, the temporal turnover of species slowed down, and communities became more similar through time. However, in saturated communities, increasing birth rates expanded ASA that then had little effect on species composition as the communities grew asymptotically toward saturation in terms of individuals (Figure 3). In this case, community composition was mainly dominated by locally produced offspring. Since the immigration rates were fixed, immigrants from the regional species pool contributed little to compositional change. This pattern is linked to the dilution effect (Kadmon & Benjamini, 2006), where the ratio between locally produced individuals and immigrants influence the number of species that coexist in a given community.

What effect does disturbance have on the relationship between species turnover and ASA? The results showed that disturbed communities experienced faster species turnover than undisturbed communities (Figure 3). Disturbance caused increased mortality among seedlings, reduced ASA, reduced total number of individuals, and prevented communities reaching saturation in terms of individuals (Figure 1a). This finding is consistent with a previous study showing that disturbed forests experienced a greater turnover of functional traits than expected (Swenson et al., 2012). When the between‐disturbance intervals were larger than one, for comparisons between disturbed communities and ones closely followed (*t*_0_–*t*_1_ for the case of disturbance frequency equal to 3 years, and *t*_0_–*t*_1_ and *t*_0_–*t*_2_ for the case of disturbance frequency equal to 5 years), disturbance also caused the asymptotic relationship between community similarity and ASA to become unimodal (Figure 3b,d,e); this unimodal curve showed community similarity decreasing as ASA increased. In disturbed communities, as in undisturbed communities, there existed an initial positive phase between ASA and community similarity (Figure 3). This initial phase suggests that stochastic extinctions were becoming less likely as ASA increased (McGlynn et al., 2010; Srivastava & Lawton, 1998). From this, we can conclude that, regardless of disturbance, demographic stochasticity is the main driver of species turnover at low ASA. But it was the emergence of the negative, decreasing phase in strongly disturbed communities that we found most interesting, attributing this to the influence of disturbance on seedling mortality. While increasing the birth rates of adults led to an increased number of seedlings and larger communities, disturbance had relatively minor influence on species richness (Figure 1b). In our simulations, we assumed that disturbance only increased the mortality rate of seedlings. This implied that for a given species even if all seedlings were killed by disturbance, the survival of its adults maintains species’ persistence in communities, which explained the minor change of disturbance on species richness. Thus, we could speculate that similarity indices only considering presence–absence information would have a lower probability to detect the decreasing trend between ASA and compositional change. Simulated communities with larger ASA had more seedlings than ones with smaller ASA. When disturbance occurred, more seedlings in disturbed communities with larger ASA were killed and left more vacant space for new seedlings (offspring of local adults and immigrants from regional pool), which led to faster turnover measured by the Bray–Curtis similarity index taking account of species abundance (Figure 3b,d,e). This legacy effect of disturbance gradually waned over time away disturbance events (Figure 3c,f,g).

Many empirical studies of heavily disturbed plant communities have attempted to explore the causes and consequences of disturbance on population dynamics and community structure (Sukumar et al., 2005; Suresh et al., 2010). For instance, in the Mudumalai permanent forest dynamics plot, the causes of disturbance in this region were categorized into three groups: Death of the aboveground stems caused by fire, herbivory by elephants, and other natural causes including the effects of drought, windfall, and disease (Suresh et al., 2010). It has been demonstrated that the effects of these types of disturbance on demographic rates are strongly stage dependent (Suresh et al., 2010). In other words, smaller individuals suffer more than larger individuals, especially under elephant herbivory. Suresh et al. (2010) attributed the mortality of small to medium woody stems in this forest plot mostly to these factors. In line with our simulations of strongly disturbed communities, empirical evidence from the Mudumalai plot confirms that increasing ASA increases rather than decreases species turnover and compositional similarity. These results contrast with the undisturbed simulations.

Although we accounted for just two stages (seedlings and adults) in the model, our stage‐structured simulations captured the essential demographic effects of disturbance on individuals in many natural communities where seedlings suffer more from disturbance than adults. Potential refinements to our model would be to: (1) Incorporate species‐specific responses to disturbance through the variability of demographic rates between species, to release the assumption of neutrality of our model (Walker, Lodge, Guzmín‐Grajales, & Fetcher, 2003), (2) Explicitly simulate the growth of individuals resulting in continuous size distribution rather than two discrete stages in the present work, and (3) Explore the impact of different types of stage‐dependent responses to disturbance, such as the scenario of adults suffering more than seedlings in terms of extreme drought (Bennett, McDowell, Allen, & Anderson‐Teixeira, 2015; Meakem et al., 2017).

## CONFLICT OF INTEREST

None declared.

## AUTHOR CONTRIBUTIONS

YW, SW, and CC conceived and designed the study and wrote the manuscript with input from MDFE and ADM contributed.

## Supporting information


** **
