# Supplementary material for: Temporal effects of disturbance on community composition in simulated stage‐structured plant communities
Source: Ecol Evol. 2017 Nov 23;8(1):120–7. doi: 10.1002/ece3.3660 (PMC5756851; doi:10.1002/ece3.3660)
Supplement: Supplementary file 1 [file ECE3-8-120-s001.docx]

**Supporting Information**


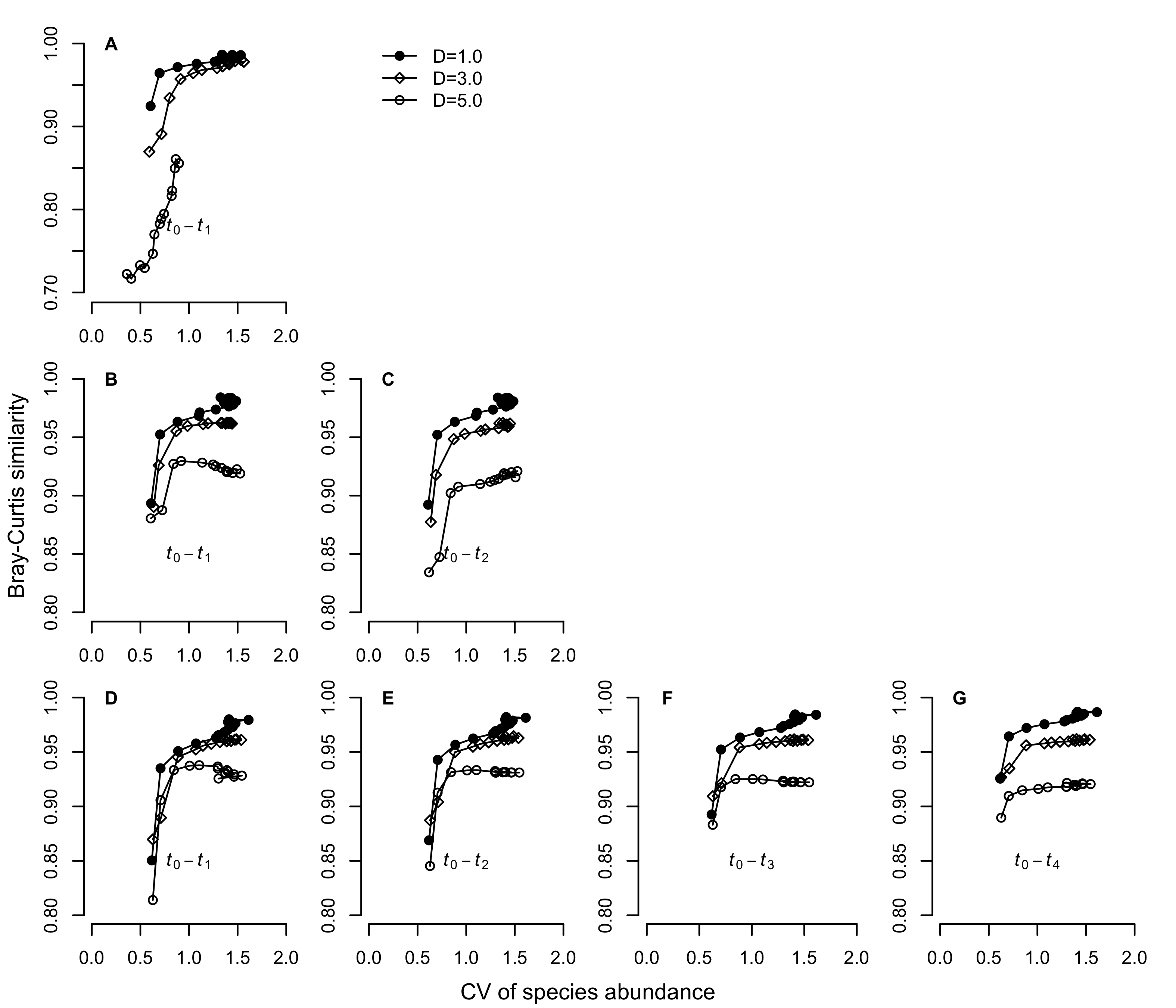


**Figure S1.** Relationships between CV of species abundance and Bray-Curtis similarity values from simulations with the immigration rate equal to 60. (A) Relationships between CV of species abundance and Bray-Curtis similarity values for the case with the disturbance frequency equal to one. (B-C) Relationships between CV of species abundance and Bray-Curtis similarity values for the case with the disturbance frequency equal to three years. (D-G) Relationships between CV of species abundance and Bray-Curtis similarity values for the case with the disturbance frequency equal to five years. Three levels of disturbance intensity were explored: *D* = 1.0, 3.0, and 5.0. The maturity time for seedlings was equal to three years. The labels of *t*_0_-*t*_1_, *t*_0_-*t*_2_, *t*_0_-*t*_3_, and *t*_0_-*t*_4_ represent the comparisons between the disturbed community (*t*_0_) and the communities one, two, three and four years after a disturbance event. Each data point represents the mean of ten replicates for each parameter combination.

**
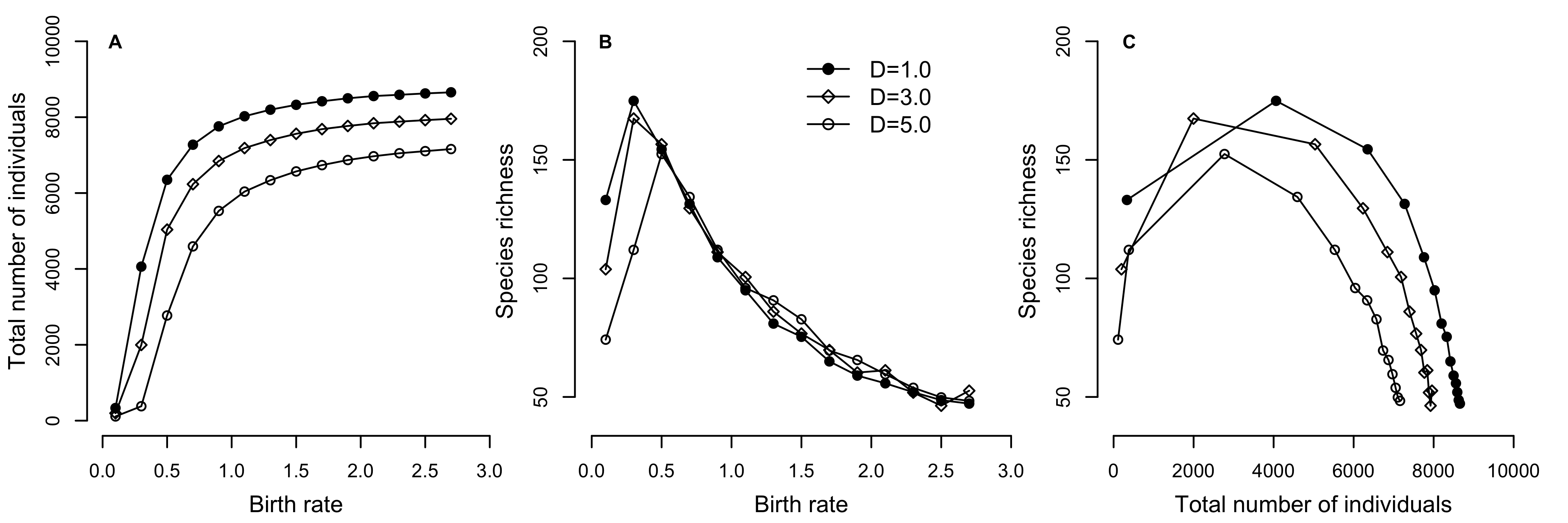
**

**Figure S2.** Influence of birth rate on community metrics with the immigration rate equal to 20. (A) Simulated relationships between birth rate and the total number of individuals, (B) Simulated relationships between birth rate and species richness, and (C) Simulated relationships between the total number of individuals and species richness. Three levels of disturbance intensity were explored: *D* = 1.0, 3.0, and 5.0. *D* = 1.0 means no disturbance. The case with the disturbance frequency equal to two years was presented as an example here. Each data point represents the mean of ten replicates for each parameter combination.

**
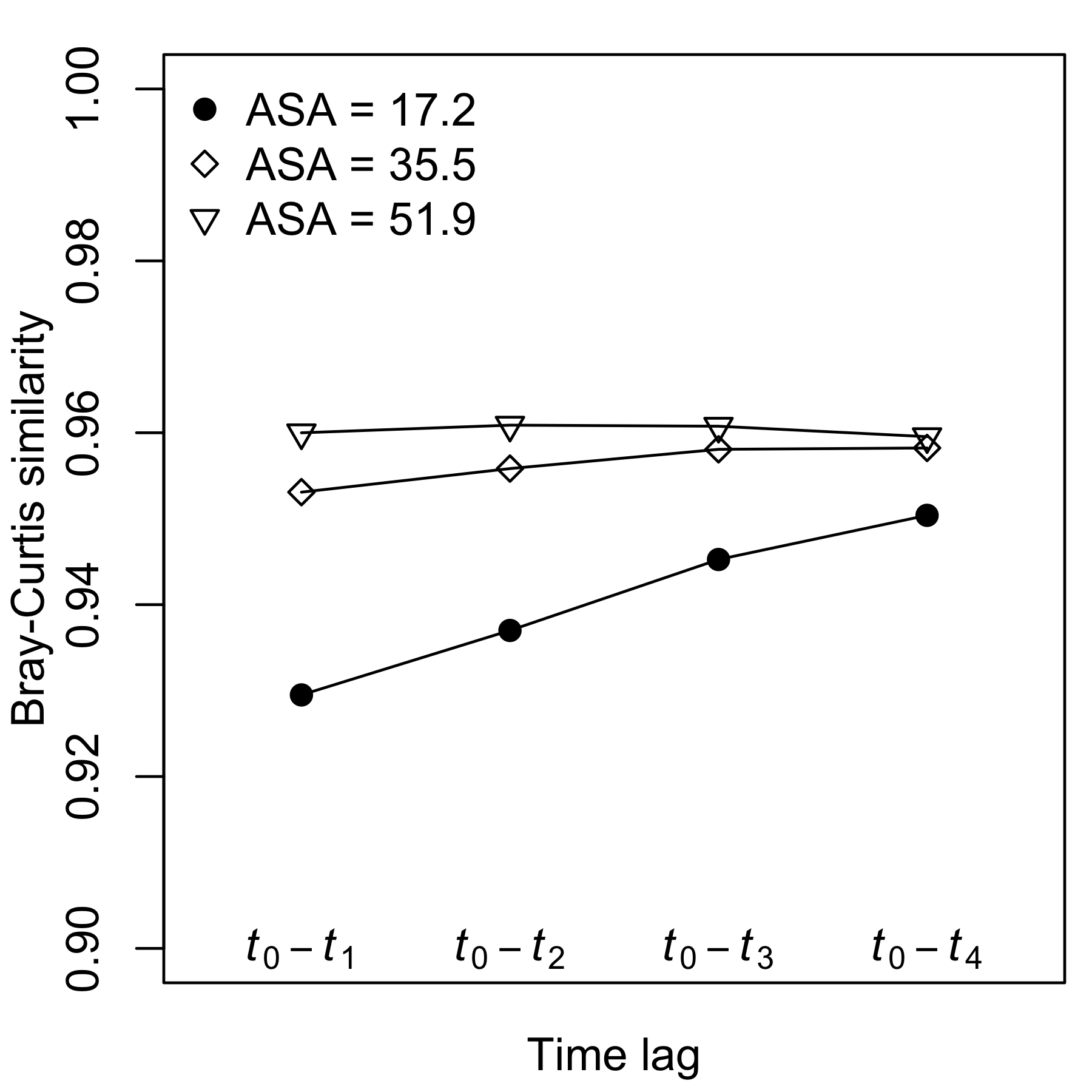
**

**Figure S3.** Compositional change of simulated communities over time with the immigration rate equal to 20. The case with the disturbance frequency (*F*) equal to five years and disturbance intensity (*D*) equal to three was presented as an example here. ASA represents average species abundance determined by demographic processes as a result of disturbance. The labels of *t*_0_-*t*_1_, *t*_0_-*t*_2_, *t*_0_-*t*_3_, and *t*_0_-*t*_4_ represent the comparisons between the disturbed community (*t*_0_) and the communities one, two, three and four years after a disturbance event. Each data point represents the mean of ten replicates for each parameter combination.

**
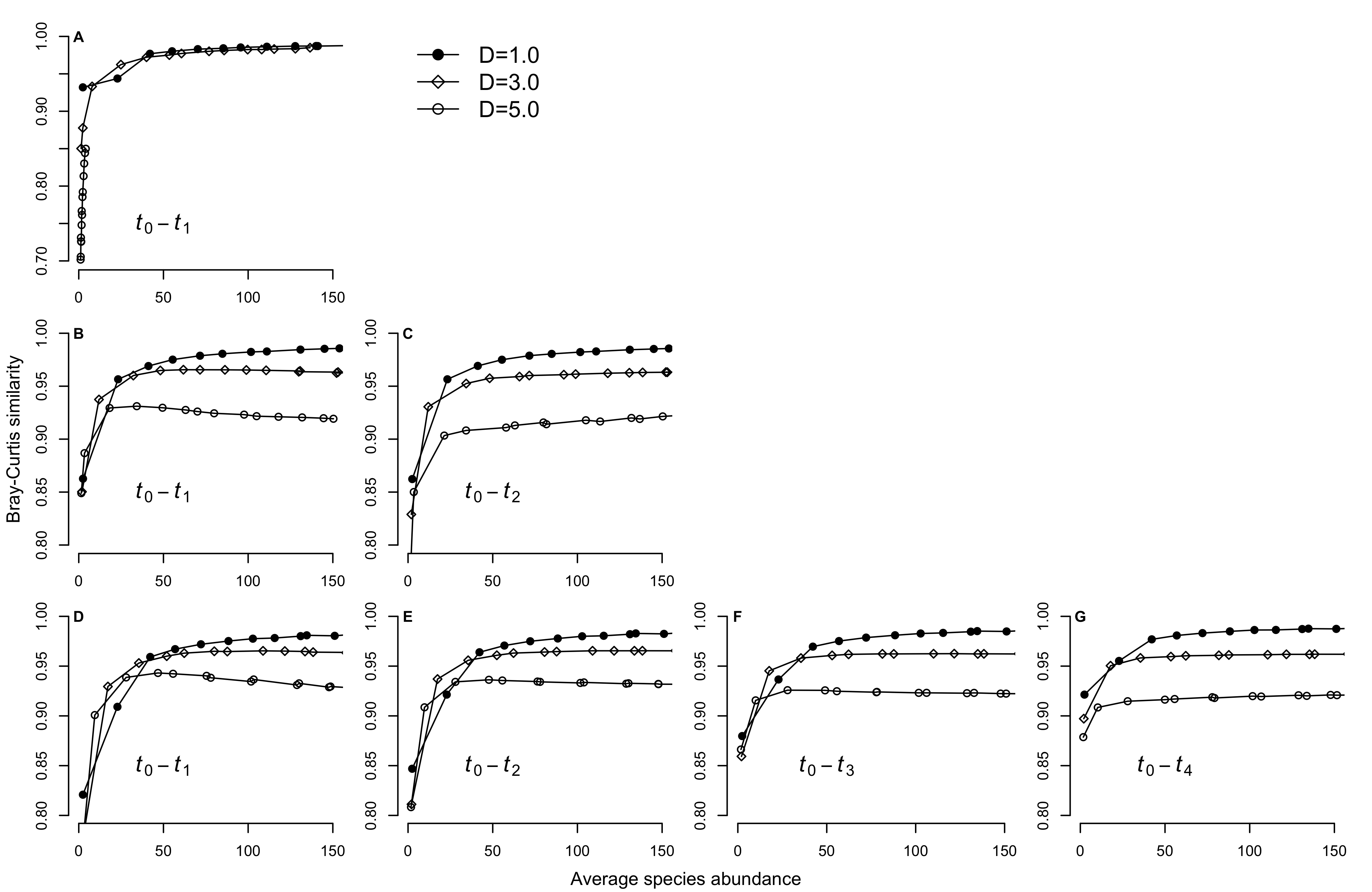
**

**Figure S4.** Simulated patterns between average species abundance and temporal compositional change with the immigration rate equal to 20 measured by Bray-Curtis similarity (A, the disturbance frequency equal to one year; B-C, the disturbance frequency equal to three years; D-G, the disturbance frequency equal to five years). Three levels of disturbance intensity were explored: *D* = 1.0, 3.0, and 5.0. The maturity time for seedlings was equal to three years. The labels of *t*_0_-*t*_1_, *t*_0_-*t*_2_, *t*_0_-*t*_3_, and *t*_0_-*t*_4_ represent the comparisons between the disturbed community (*t*_0_) and the communities one, two, three and four years after a disturbance event. Each data point represents the mean of ten replicates for each parameter combination.
